# Granzyme M expressed by tumor cells promotes chemoresistance and EMT *in vitro* and metastasis *in vivo* associated with STAT3 activation

**DOI:** 10.18632/oncotarget.3461

**Published:** 2015-02-28

**Authors:** Huiru Wang, Qing Sun, Yanhong Wu, Lin Wang, Chunxia Zhou, Wenbo Ma, Youhui Zhang, Shengdian Wang, Shuren Zhang

**Affiliations:** ^1^ Department of Immunology, Cancer Hospital & Cancer Institute, Peking Union Medical College & Chinese Academy of Medical Sciences, Beijing, China; ^2^ Department of Parasitology, Capital Medical University, Beijing, China; ^3^ Department of Pathology, Cancer Hospital & Cancer Institute, Peking Union Medical College & Chinese Academy of Medical Sciences, Beijing, China; ^4^ Center of Infection and Immunity, Institute of Biophysics, Chinese Academy of Sciences, Beijing, China

**Keywords:** cancer, granzyme M, chemoresistance, metastasis, epithelial-mesenchymal transition

## Abstract

Granzyme M is a serine protease known to be often expressed by natural killer cells and induce target cells apoptosis in combination with perforin. However, we detected granzyme M expression in murine and human cancer cell lines and human tumor samples in our study. Granzyme M increased chemoresistance, colony-formation, cytokine secretion and invasiveness *in vitro*. Most importantly, granzyme M facilitated tumor growth and metastasis *in vivo*. Granzyme M induced the epithelial-mesenchymal transition (EMT) in cancer cells associated with STAT3 activation. Our study revealed the role of granzyme M expressed by tumor in chemoresistance, invasion, metastasis and EMT.

## INTRODUCTION

Despite the marked progress that has been made by numerous oncologists in the battle against cancer over the past decades, drug resistance, metastasis and relapse resulting in high mortality remain a huge challenge to clinical therapy [1]. Our previous study demonstrated that slow-cycling tumor cells are more resistant to chemotherapy and may be the source of tumor relapse and metastasis. We expect to find some new clues that account for tumor metastasis and chemoresistance through microarray assays. Accordingly, we focus on a unique gene termed granzyme M (GZMM) that was upregulated in slow-cycling tumor cells.

GZMM, which is located on human chromosome 19 and syntenic mouse chromosome 10C co-localized with a family of neutrophil elastase genes, is the only granzyme located in this cluster [2]. It is sometimes termed one of the ‘orphan granzymes’ because of our rudimentary understanding of it [3]. GZMM, as a serine protease, is often expressed in natural killer cells and can induce cell apoptosis in combination with perforin via the proteolysis of intracellular substrates [4,5]. However, the cytotoxic potential of GZMM is highly controversial due to a lack of a consensus regarding pathways and substrates that are involved *in vitro* [6,7].

The expression of GZMM, which was initially considered to be restricted to NK cells, is currently known to be more extensive [8]. For example, GZMM is found to be expressed in T-cell granular lymphocytic leukemia cells and a solid tumor cell line (Hela) [9, 10]. Similarly, granzyme B, another typical granzyme, was also detected in human primary breast carcinomas, primary bladder cancers and pancreatic carcinoma cells [11-13]. Moreover, it is gradually clear that multitudinous proteases, including matrix metalloproteinases (MMPs), cathepsin, and the urokinase-type plasminogen activator (uPA) system, participate in degrading extracellular matrix (ECM) elements during invasion and metastasis in malignant tumor progression [14-16]. Consequently, we were encouraged by these findings to investigate the expression of GZMM in solid tumor cells and its function in cancer progression.

In the present study, we took a directed approach to explore the expression and function of GZMM in cancer cells for the first time. We found that GZMM is expressed in common murine carcinoma cell lines, human cancer cell lines and clinical carcinoma samples, which largely expand our knowledge of this so-termed “orphan” granzyme. In murine tumor cell models, GZMM can lead to heightened chemoresistance, increased cytokine release, augmented invasion *in vitro* and enhanced metastases and tumor growth *in vivo*. All of these changes are related with the EMT process and STAT3 activation.

## RESULTS

### GZMM is expressed in murine and human malignant cell lines

**Figure 1:**
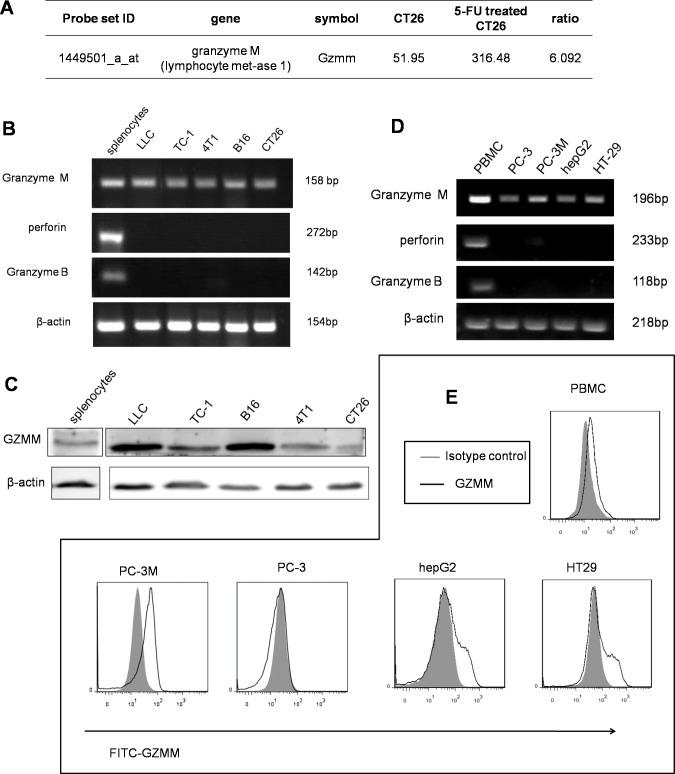
GZMM is expressed in murine and human malignant cell lines (A) The microarray results showed that GZMM was significantly upregulated in slow-cycling CT26 cells compared with the control. (B&D) GZMM, perforin and GZMB were measured in murine and human tumor cell lines by RT-PCR using lymphocytes as the positive control. β-actin was included as an internal control. (C) Western blot analyses of the protein levels of GZMM in murine tumor cell lines. β-actin was included as an internal control. (E) GZMM expression by human tumor cell lines and PBMC were determined by flow cytometry.

In a previous study, we demonstrated that slow-cycling murine colon carcinoma cells (CT26) exhibited the character of cancer stem cells, such as resistance to chemotherapeutics and heightened tumor initiation [17]. Through microarray gene expression analyses, we identified several pathways that are critical for cancer survival, chemoresistance, angiogenesis and inflammation (Figure S1). Moreover, we found that GZMM was significantly upregulated in slow-cycling CT26 cells compared with the control (Figure 1A).

To explore whether the expression of GZMM in tumor cells is universal, we validated the expression of GZMM at both the mRNA and protein levels in several murine tumor cell lines using Balb/c lymphocytes as the positive control. RT-PCR analysis revealed the expected fragment of GZMM (SinoGenoax) in 4T1, CT26, B16, LLC, and TC-1 cells, as same as the fragment derived from splenocytes. However, both perforin and granzyme B, which are thought to be functionally related to GZMM, were not detected by RT-PCR in murine tumor cell lines (Figure 1B). A western blot analysis revealed that the GZMM protein was also present in those tumor cells (Figure 1C).

We also validate the expression of GZMM in human malignant cells include HT29, HepG2, PC-3 and PC-3M cells, with human peripheral blood mononuclear cells as the positive control. Similarly, mRNA expression was also confirmed in murine tumor cells while perforin or granzyme B was not detected (Figure 1D). As the flow cytometry results shown, GZMM was also expressed in the cytoplasm of PC-3M, HepG2 and HT29 but not in PC-3 (Figure 1E). Therefore, we speculated that the possible function of GZMM may not be restricted to cytotoxicity. It is notable that PC-3M, as a subline with the high metastatic potential of the prostate cancer cell line PC-3, maintained a higher expression of GZMM than PC-3. These observations suggest that GZMM was not only expressed by immune cells but also by neoplastic cells independent of perforin.

### GZMM is abnormally overexpressed in clinical carcinoma samples

**Figure 2:**
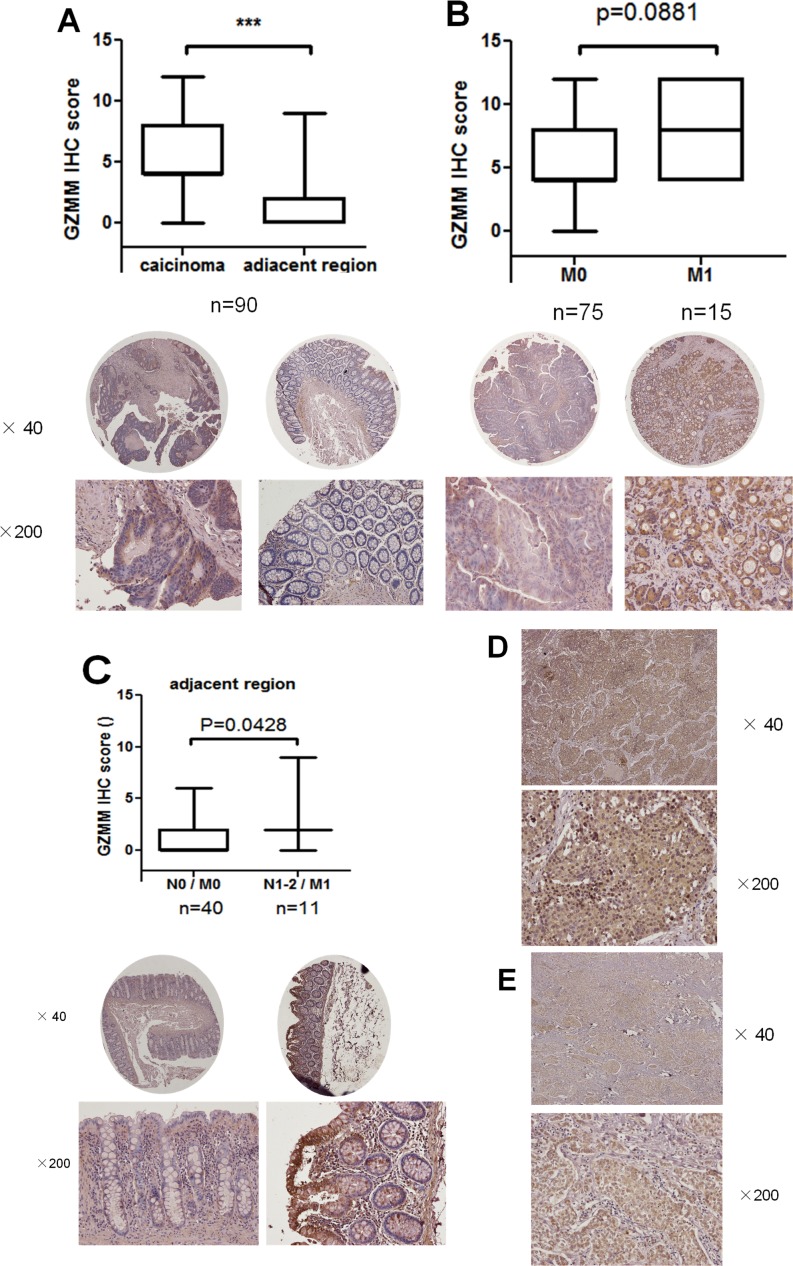
GZMM is abnormally overexpressed in clinical carcinoma samples (A) GZMM staining scores of primary 90 colon carcinoma samples and matching adjacent regions were displayed. Representative images were displayed at the bottom. ***p<0.001. (B) GZMM staining scores of 75 carcinoma samples without metastasis(M0) and 15 samples with metastases (M1) were shown. Representative images were displayed at the bottom. (C) GZMM staining scores of peritumoral regions of 40 colon cancer patients without metastasis or lymphatic metastasis (N0 / M0) and 11 patients with metastases and lymphatic metastases (N1-2 / M1) were shown. Representative images were displayed at the bottom. (D&E) Representative images of immunohistochemical staining of GZMM in lung adenocarcinoma (D) and breast cancer samples (E) were displayed. The IHC score was performed by pathologists and shown by box-and-whisker plots.

We next analyzed the expression of GZMM by immunohistochemistry staining in 90 surgical samples of human primary colon carcinoma and matching normal regions adjacent to the tumor (Figure S2). The results revealed GZMM was abnormally expressed in most carcinoma cells but rarely in adjacent normal epithelial cells (Figure 2A). Nonetheless, there is no positive correlation between the expression level of GZMM and the histopathological grade of colon adenocarcinoma (data not shown). We also investigated the relationship between the expression of GZMM and metastases and found the average IHC scores of GZMM in 15 patients with metastasis were higher than in 75 patients without metastasis. Unfortunately, the change was not significant maybe due to the limited cases (P=0.0881, Figure 2B). However, IHC scores of GZMM in normal regions adjacent to the tumor of 11 patients with metastasis and lymphatic metastasis were significantly higher than that without metastasis or lymphatic metastasis, as shown in (Figure 2C).

In addition, in clinical samples of lung adenocarcinoma and breast cancer, GZMM expression was also identified (Figure 2D and Figure 2E). These results indicate that GZMM was universally expressed by clinical malignant cells and may be correlated with metastasis and lymphatic metastasis.

### GZMM enhanced chemoresistance in murine tumor cells

**Figure 3:**
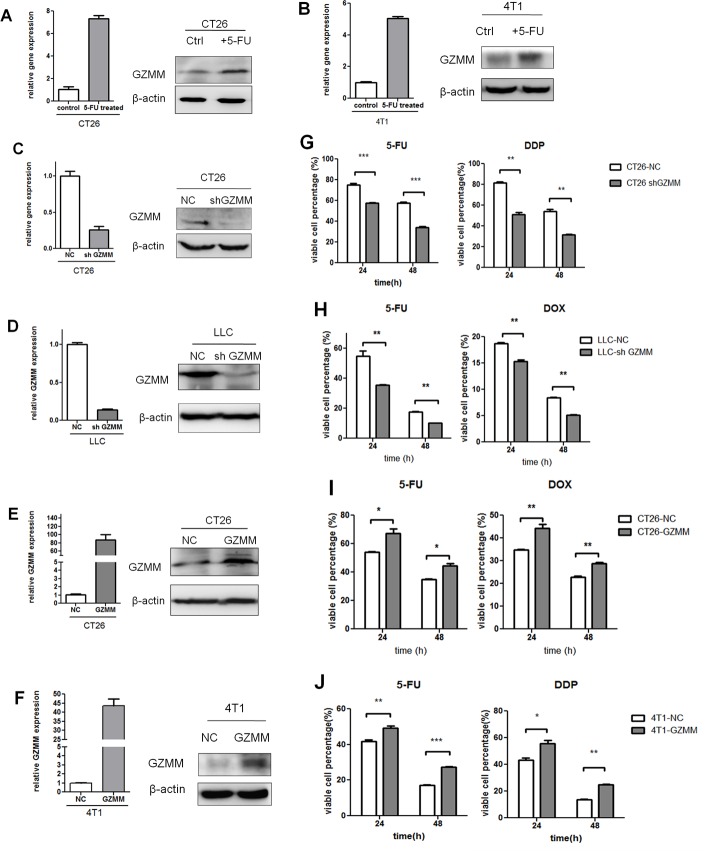
GZMM enhances chemoresistance in murine tumor cells (A-B) The mRNA and protein levels of GZMM in 5-FU-treated (2 μg/mL, as we described previously) CT26, 4T1 and control cells were analyzed by qRT-PCR and western blot. (C-D) Efficiency of stable GZMM knockdown in CT26 and LLC were determined by qRT-PCR and western blot. (E-F) Efficiency of GZMM overexpression in CT26 and 4T1 were shown. β-actin was included as an internal loading control. (G) CT26-NC, CT26-sh GZMM, (H) LLC-NC, and LLC-sh GZMM cells were treated with 5-FU (2 μg/ml), DDP (2 μg/mL) or doxorubicin (2 μg/ml) and control medium. After 24 or 48 hours, the variable cell percentage was determined by CCK-8 assay. (I) CT26-NC, CT26-GZMM were treated with 5-FU (4 μg/ml), doxorubicin (2 μg/ml), 4T1-NC, 4T1-GZMM were treated with 5-FU (2 μg/ml) or DDP (2 μg/mL) and control medium. After 24 or 48 hours, the variable cell percentage was determined by CCK-8 assay. The experiments were repeated three times with similar results. *p<0.05, **p<0.01. n=3. The data are presented as the means ± SEM.

Since the microarray results indicate GZMM was upregulated in 5-FU-treated CT26 cells, we validate it by Quantitative RT-PCR and western blot in CT26 and 4T1 cells. As revealed in (Figure 3A and Figure 3B), after 5-FU Treated, GZMM was indeed aberrantly elevated.

To identify whether the expression of GZMM plays an important role in the maintenance of chemoresistance, we infected 4T1 and CT26 cells with lentivirus to generate cells that stably express the empty vector and the cDNA of murine GZMM, and infected LLC and CT26 cells to generate cells that were stably knockdown of GZMM and control cells. Quantitative RT-PCR and western blot were used to confirme the efficiency of gene transduction and protein expression (Figure 3C-3F). These cells were tested to determine their capacity of proliferation and sensitivity to chemotherapeutics *in vitro*. As shown in Figure S3, GZMM had almost no significant influence on the proliferation of 4T1, LLC, CT26 cells. 5-FU, Doxorubicin (DOX) and cis-platinum (DDP), as several types of common chemotherapeutics, were applied to assess the role of GZMM in the resistance to chemotherapeutics. As the results shown in (Figure 3G-3J), GZMM knockdown enhanced the sensitivity to cytotoxicity due to chemotherapy than the control, whereas GZMM overexpression promoted the survival of cells undergoing treatment with chemotherapeutics. Thus, these results indicated that the expression of GZMM can enhance the resistance to common chemotherapeutics.

### GZMM promotes colony forming capacity, invasiveness and cytokine secretion *in vitro*

**Figure 4:**
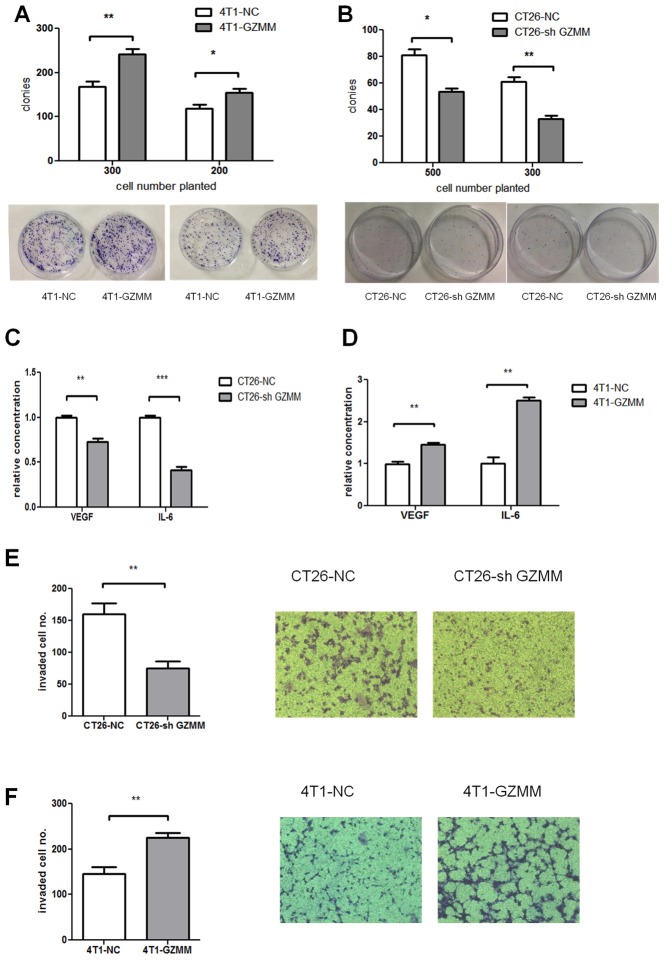
GZMM promotes cloning forming capacity, invasiveness and cytokine secretion *in vitro* (A-B) 300 or 500 cells were seeded in 60mm-plates in quadruplicate with complete medium and cultured for one week at 37°C. After cultured, cells were fixed, stained with Giemsa and counted as shown. (C-D) The secreted murine VEGF and IL-6 protein levels in the supernatants of CT26-NC, CT26-sh GZMM, 4T1-NC, and 4T1-GZMM cells were measured by an ELISA kit. (E-F) The cells were seeded in the upper chamber with matrigel. After incubation for 20 h, the cells that migrated to the lower surface of the membrane were counted, as shown in the figure. The data are expressed as the mean values ± SEM from one experiment performed in triplicate from three independent experiments. **p<0.01, ***p<0.001.

Since GZMM seldom influence the proliferation of tumor cells, we attempted to exam the effect on cloning forming. As the (Figure 4A and Figure 4B) shown, 4T1-GZMM could form more visible colonies than the control cells, while less colonies formed by CT26-sh GZMM compared with counterparts. This observation could explain GZMM enhanced chemoresistance via heightening clone formation in some degree.

Tumor-promoting inflammation and angiogenesis play a significant role in the development and progression of a variety of animal and human cancer models, as indicated by epidemiological and experimental evidence. Therefore, we performed ELISA for several types of cytokines released from *in vitro*-cultured CT26-sh GZMM, CT26-NC, 4T1-NC and 4T1-GZMM. The results revealed a significant decrease in interleukin-6 (IL-6) and vascular endothelial growth factor (VEGF) upon CT26-sh GZMM treatment compared with the control, whereas 4T1-GZMM produced more VEGF and IL-6 than its counterparts (Figure 4C-4D). We observed the relationship between GZMM expression and the secretion of IL-6 and VEGF from cancer cells for the first time.

Extracellular matrix degradation by proteolysis is critical for tumor invasion and metastasis [15]. We hypothesized that GZMM, as a serine protease, may promote tumor invasion by hydrolyzing its substrates contained in matrigel. To investigate whether the expression of GZMM enhances tumor invasion, we used a transwell chamber with 3-mm-thick matrigel. As revealed in (Figure 4E and Figure 4F), the number of CT26-sh GZMM cells invading through the matrigel to the lower chamber was markedly decreased compared with the control cells after 20 hours of incubation, whereas the 4T1-GZMM cells showed to be more invasive than the counterparts. These results provide the first evidence of GZMM promoting tumor invasion *in vitro*.

### GZMM facilitates tumor growth and metastasis *in vivo*

**Figure 5:**
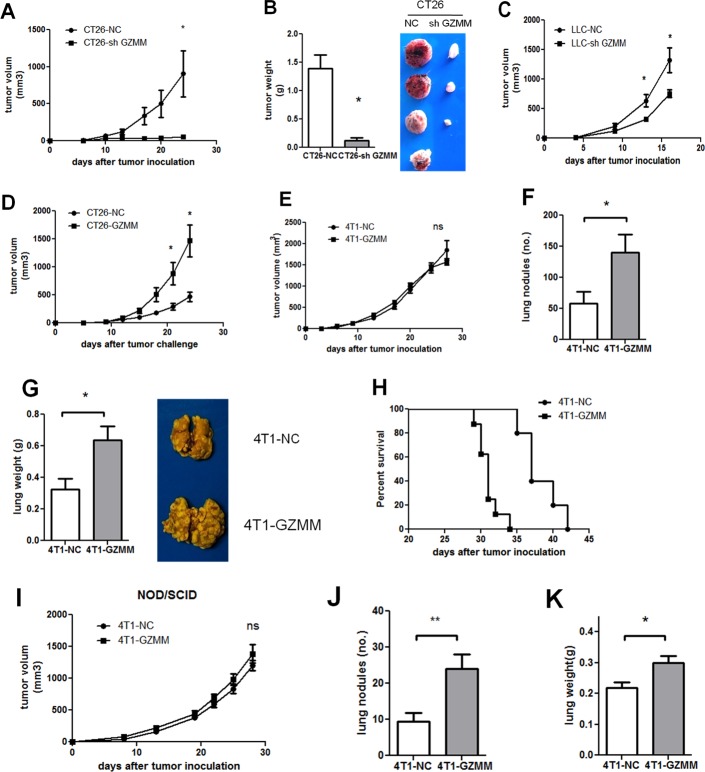
GZMM facilitates metastasis and tumor growth *in vivo* (A-B) The primary tumor volumes and tumor weight of BALB/c mice bearing CT26-NC and CT26-sh GZMM (2×10^5^) were shown (four mice per group, one mouse bearing CT26-sh GZMM did not form a tumor). (C) The primary tumor growth of BALB/c mice bearing LLC-NC and LLC-sh GZMM (4×10^5^) were displayed (five mice per group). (D) The primary tumor growth of BALB/c mice bearing CT26-NC and CT26-GZMM (1×10^5^) were shown (five mice per group). (E) The primary tumor growth of BALB/c mice bearing 4T1-NC and 4T1-GZMM (1×10^5^) are shown. (F-G) Four weeks after tumor inoculation, the mice were sacrificed, and the lung metastatic nodules and the lung weight were measured. Each group contained five mice. (H) The graph represents the Kaplan-Meier survival analysis of BALB/c mice baring 4T1-NC and 4T1-GZMM (seven to eight mice per group, P=0.0008). (I) The primary tumor growth of NOD/SCID mice grafted with 4T1-NC or 4T1-GZMM cells (1×10^5^) were shown (seven to eight mice per group). (J-K) Four weeks after tumor inoculation, the mice were sacrificed, and the lung metastatic nodules and lung weights were measured. The data are expressed as the mean values ±SEM from one experiment performed from three independent experiments. *p<0.05.

To assess the role of GZMM *in vivo*, the CT26-knockdown mouse model was used to estimate tumor growth. As shown in (Figure 5A and Figure 5B), tumor-bearing CT26-sh GZMM was markedly inhibited compared with the control group. Although it appears inconsistent with the *in vitro* features, this finding may indicate that *in vivo* tumor growth is regulated by the many complicated components in the tumor microenvironment. Likewise, knockdown of GZMM in LLC delayed the tumor growth in C57 mice as (Figure 5C) displayed. Moreover, overexpression of GZMM in CT26 accelerated tumor growth compared with the control (Figure 5D).

Because the invasive potential of GZMM was validated *in vitro*, a subcutaneous 4T1 mouse model was established to assess the role of GZMM in tumor invasion and metastases *in vivo* by surveying the pulmonary metastases. Consistent with the proliferation *in vitro*, we observed no significant difference in the growth of primary tumor between 4T1-GZMM and control cells starting from the time of tumor cell injection to the end of the experimental period (Figure 5E). In contrast, the mice bearing 4T1-GZMM developed significantly increased numbers of lung tumor metastatic nodules and multiple tumor fusions compared with the control mice bearing 4T1-NC (Figure 5F and Figure 5G), which indicated a larger tumor burden in the lung of mice bearing 4T1-GZMM relative to the control. Moreover, heart and liver metastases were also found in the mice bearing 4T1-GZMM, whereas none were found in the control mice.

Because tumor metastasis exhibits a large risk of mortality, we also monitored the survival of tumor-bearing mice. As expected, the survival of mice bearing 4T1-GZMM was notably shorter compared with the control mice (median survival of 37 versus 31 days, as shown in Figure 5H). All of these observations implied the considerable function of GZMM in facilitating cancer cell metastasis *in vivo*.

This effect was also validated in immune deficiency mice. Similarly, the primary tumor growth in NOD/SCID mice was in accordance with that found in immune competent mice (Figure 5I). Nevertheless, the finding revealed that pulmonary metastases of 4T1-GZMM were approximately twofold higher than those found for 4T1-NC on day 28 after tumor inoculation (Figure 5J). Interestingly, we found decreased pulmonary metastases in both 4T1-NC- and 4T1-GZMM-bearing NOD/SCID mice (9.375±2.434 versus 24±3.94) compared with Balb/c mice (58.4±18.49 versus 140±28.69) (Figure 5F and Figure 5J). The data suggest that the metastasis of 4T1 may be associated with the immune system during cancer progression, and the effect of GZMM may be partially dependent on immune cells *in vivo*. Taken together, all of these results indicate that the overexpression of GZMM facilitates pulmonary metastases *in vivo*.

Therefore, all of the above-mentioned observations prove that GZMM facilitates tumor growth and metastasis *in vivo*, which provides a new clue for tumor therapy.

### GZMM is associated to the epithelial-mesenchymal transition of cancer cells

**Figure 6:**
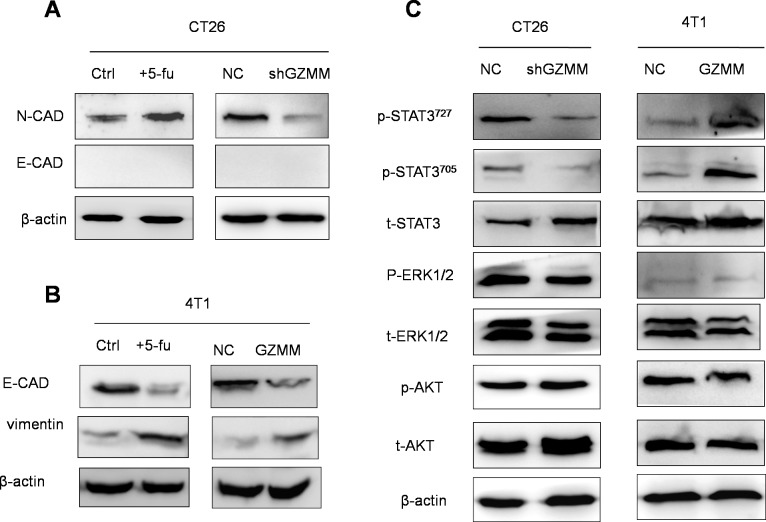
GZMM is associated with the epithelial-mesenchymal transition of cancer cells, and STAT3 signaling pathway activation is involved in GZMM resulting in the EMT (A-B) Western blot analyses of epithelial and mesenchymal markers (E-cad, N-cad, and vimentin) in 4T1/CT26 cells and 5-FU-treated counterparts, i.e., 4T1-NC/CT26-NC and 4T1-GZMM/CT26-sh GZMM, are shown. (C) The total AKT, ERK1/2, and STAT3 and the protein phosphorylation level in 4T1-NC, 4T1-GZMM, CT26-NC and CT26-sh GZMM cells were determined by western blot, as displayed in the figure. β-actin was included as the sample loading control.

In cancer cells, the epithelial-mesenchymal transition (EMT) process confers migratory and invasive capacity, resistance to apoptosis, drug resistance, evasion of host immune surveillance, and tumor stem cell traits [18]. Cells undergoing the EMT may represent tumor cells with metastatic potential. As shown in (Figure 6A and Figure 6B), slow-cycling CT26 treated with 5-FU exhibit more s (N-cad) than the control. In addition, 4T1 displayed less epithelial features (E-cad) and more mesenchymal characteristic (vimentin) after 5-FU treatment. To investigate whether EMT was responsible for the process of GZMM promoting drug resistance, invasion and metastasis, we explored the expression of epithelial and mesenchymal markers in CT26-sh GZMM, 4T1-GZMM and their control cells. The findings show that GZMM upregulation was accompanied by less epithelial features in 4T1, whereas GZMM downregulation occurred concurrently with decreased mesenchymal characteristics in CT26. These observations led to the hypothesis that the expression of GZMM in tumor cells is likely to be correlated with the EMT.

### STAT3 signaling pathway activation is involved in GZMM resulting in the EMT

Abnormal signaling pathway activation, including AKT, IL-6/STAT3 and MAPK/ERK, is considered to be significant for the EMT, pro-tumorigenic inflammatory cytokine secretion, invasion and metastasis of tumor cells [19-21]. Therefore, to address which signaling pathway is involved in this process, the phosphorylation levels of AKT, STAT3 and ERK were monitored by western blot, and we found that the phosphorylation levels of AKT and ERK did not change markedly. However, STAT3 phosphorylated at both Try705 and at Ser727 were elevated markedly in 4T1 cells overexpressing GZMM and decreased in CT26 cells in which GZMM is downregulated, whereas the total STAT3 levels remained unchanged, indicating that GZMM may influence the activation of STAT3 resulting in characteristic changes in CT26 and 4T1 cells (Figure 6C). This finding is also in accordance with the enhancement of IL-6 and VEGF described above because a high phosphorylation level of STAT3 has been demonstrated to play a predominant role in the regulation of transcription activation immunosuppressive cytokines, including IL-6 and VEGF [22]. Taken together, these results indicate that the activation of STAT3 is closely related with the advancement of invasion and metastasis driven by GZMM resulting in the EMT.

## DISCUSSION

It is now generally accepted that metastasis causing mortality is the prominent challenge in tumor research and clinical therapy. A great number of complicated elements have been implicated in the degradation of the basement membrane and separation from primary tumor and thereby facilitation of metastasis. Among all of the proteases involved in this process, the best-studied are the MMPs, which can degrade the extracellular matrix, increase the inflammatory response and modify signaling pathways via precise proteolytic processing [23]. Granzyme, e.g., granzyme B, which belongs to a set of serine proteases of cytotoxic lymphocytes, combined with perforin is considered the key effector of immune reaction. However, an increasing body of evidence indicates that granzyme functions diversely, including the stimulation of cytokine secretion, extracellular matrix degradation, growth factor release and direct antiviral effects [24-28].

We previously detected murine GZMM expression in the murine colon cancer cell line CT26 through microarray gene expression analysis. To assess whether the expression of GZMM is dependent on a specific type of cancer cell line, we performed the measurement and found that the expression of GZMM is conserved in several types of murine and human malignant cell lines. Similarly, GZMM was found to be expressed in human the cervical cancer cell line HeLa at both the mRNA and protein levels by chance, thus, its function was not well investigated [10]. Coincidentally, granzyme B, as another typical granzyme, was also found to be expressed in human urothelial carcinoma samples and pancreatic cancer and to be associated with the tumor epithelial-mesenchymal transition and invasion *in vitro*. Most of the studies on GZMM have reported its cytotoxicity, but there is no agreement on the substrates and pathways that are involved in this process [6]. We hypothesized that the reason may be related with the lack of a recognized standard method for the measurement of granzyme activity and substrates. Some other studies found that murine GZMM is closely involved in the inflammatory response rather than the cytotoxicity of lymphocytes *in vivo*, which provides insights into the role of GZMM in tumors because it may be associated with the pro-inflammatory reaction.

In this study, in CT26 and 4T1 cells, we found that GZMM is positively related to IL-6 and VEGF release, which indicates that GZMM may be involved in tumor-associated inflammatory response and angiogenesis. Moreover, GZMM was proven to enhance chemoresistance (5-FU, cis-platinum and doxorubicin), which is in accordance with the elevated expression of GZMM in slow-cycling CT26 cells that are more resistant to chemotherapy [17]. Overexpression of GZMM facilitating colony formation may partially explain that, however, the underlying mechanism still requires further study.

We then focused on the impact of GZMM on tumor invasion and metastases because the major mortality of cancer patients is due to distant metastases. A transwell invasion model displayed that GZMM can facilitate the degradation of matrigel. We hypothesized that GZMM participates in proteolysis via specific motif cleavage. For instance, the small leucine-rich proteoglycans (SLRP) family may be hydrolyzed by GZMM due to leucine cleavage preference [29]. It remains unclear which specific motif of GZMM is cleaved to facilitate invasion, which requires a mature method for measuring granzyme activity with a specific inhibitor in the future. Nevertheless, mice bearing 4T1-GZMM presented markedly more intense metastases and poor survival, which demonstrate that a new element is involved in metastases and indicate a new target for metastatic inhibition.

Following the mechanistic investigation, the findings showed that cancer cells with a higher level of GZMM exhibited more mesenchymal features rather than epithelial cells, indicating that GZMM facilitates the EMT, which confers cells with invasive and metastatic potential, chemoresistance and immune evasion [18]. With respect to the signaling pathway related to the process of EMT, we found that the STAT3 signaling activity was markedly enhanced. STAT3 activation is able to directly affect the expression of IL-6, which is also one of the key STAT3 activators during cancer EMT progression [30, 31]. Furthermore, Tyr^705^ phosphorylation, as a transcription factor, participates in the expression of VEGF and Bcl proteins to promote angiogenesis and anti-apoptosis [32]. Strategies overcoming EMT and inhibiting STAT3 activation may reduce chemoresistance and invasion resulted from GZMM expression [33, 34]. With regard to whether GZMM induces EMT dependent on STAT3 activation as a transcription factor or via other unknown models, it is necessary to confirm these findings through subsequent research.

We also attempted to explore the significance of GZMM in human cancer cells and confirmed its expression in colon cancer, lung adenocarcinoma and breast cancer samples. Moreover, the negative relation between GZMM and E-cad in PC-3 and PC-3M suggested that GZMM may be involved in the EMT and metastasis. Although our data obtained from 90 primary colon cancer samples cannot confirm a positive correlation between metastasis and GZMM expression, a larger number of clinical tumor samples, including primary and metastasis should be used and may acquire an inspiring result. Meanwhile, the question of whether the impact of endogenous GZMM in human cancer cells is similar with that observed in murine model remains unanswered. To evaluate this proposal, human cell model need to be established. The relationship between GZMM and the prognosis of patients also remain to be analyzed. If it can be validated, GZMM may be a novel target for cancer therapy with good prospects.

The following consensus has long been recognized: all granzymes are only cytotoxic and act as the bullets of immune cells. However, we accidentally detected GZMM expression in the microarray results and attempted to determine its biological significance. Thus, this study describes the expression of GZMM in several murine malignant cells, human carcinoma cells and clinical carcinoma samples and provides the first demonstration of a potentially important impact of GZMM on maintaining resistance to chemotherapeutics and promoting cancer invasion, metastasis and EMT. It appears that GZMM may be the weapon of tumor cells. In general, all of these results contribute to the understanding of the ‘orphan’ granzyme, which was previously rudimentary, and the mechanism of cancer chemoresistance, invasion and metastasis.

## METHODS

### Gene expression microarray

CT-26 cells were cultured in complete medium supplemented with 5-FU (2 μg/mL) on day 1. The old medium was replaced with fresh medium without 5-FU on day 2, and 5-FU was added on day 3 again. The cells were harvested on day 4. These cells were used to perform the experiments on slow-cycling cells and denoted 5-FU-treated CT26 cells. Control CT26 cells were cultured in complete medium without 5-FU. The total RNA of CT26 and 5-FU-treated CT26 (method described in [17]) was extracted, and expression profiling of the coding genes was performed using the Affymetrix Mouse Genome 430 2.0 platform supplied by CapitalBio Corporation. Pathway Enrichment Analysis was performed using the Molecule Annotation System V3.0.

### Cell line and cell culture

4T1 and CT26 cells were purchased from the American Type Culture Collection (ATCC) (Manassas, VA, USA). PC-3 and PC-3M were purchased from Institute of Basic Medical Sciences Chinese Academy of Medical Sciences, Cell Research Center (Beijing, China). The B16, Lewis Lung cancer (LLC), TC-1, HepG2 and HT-29 cells were maintained in our laboratory. The CT26, B16 and PC-3M cells were cultured in RPIM 1640 medium; 4T1 cells were cultured in DMEM/F12 medium and PC-3, TC-1, HepG2, HT-29 and LLC cells were cultured in DMEM medium. All of these cells were maintained in basic medium supplemented with 10% FCS and penicillin/streptomycin at 37°C in a humidified atmosphere of 5% CO_2_ (Thermo Scientific, Inc.).

### Immunohistochemical staining

Tissue microarrays containing paired primary colon carcinoma and peritumoral colonic mucosa tissue from 90 patients were constructed by Shanghai Biochip Co., Ltd. (Shanghai China). Sections of 4μm thickness were placed on 3-aminopropytriethoxysilane-coated slides for subsequent staining with anti-human GZMM (LifeSpan BioSciences) after the endogenous peroxidases were inactivated. Sections incubated with isotype-matched antibodies were used as the negative control. The secondary antibodies were HRP-conjugated goat anti-mouse or rabbit IgG.

### Mice

Female six-week-old Balb/C mice, C57 mice and non-obese diabetic/severe combined immunodeficient (NOD/SCID) mice (Vital River laboratory Animal Technology Co. Ltd., Beijing, China) were maintained under specific pathogen-free conditions.

### Generation of GZMM-knockdown or recombination and control cell lines

The GZMM-specific short hairpin RNA (shRNA) plasmid pGLV3/H1/GFP+Puro vector was purchased from Gene-Pharma (Shanghai, China) and the interference sequence target GZMM was GCACTGCTTAAGCTAGATAGA. The scramble sequence GTTCTCCGAACGTGTCACGT was used as the negative control (NC). Lentivirus particles were prepared by co-transfecting lentivirus plasmids (Invitrogen) and shRNA into 293T cells using lipofectamine 2000 (Invitrogen). After infected with lentivirus particles, CT26 and LLC cells stably expressing the specific short hairpin RNA (CT26-sh GZMM, LLC-sh GZMM) and control (CT26-NC, LLC-NC) were selected in media containing 2 μg/ml puromycin. Similarly, GZMM cDNA was synthesized and inserted into the pLVEF1a/GFP+Puro vector. 4T1 and CT26 cells stably expressing GZMM (4T1-GZMM, CT26-GZMM) and control (4T1-NC, CT26-NC) were selected by puromycin followed by lentivirus infection.

### Determination of VEGF and IL-6 production by ELISA

Tumor cells (1×10^4^) were seeded in a 96-well plate, and the supernatants were collected after 24 h. The cytokine production in the supernatants was measured by ELISA according to the manufacturer's guidelines (neoBiosciences Ltd., Beijing, China)

### *In vitro* proliferation and drug sensitivity assay

The CCK-8 (cell counting kit-8, DOJINDO, Japan) assay was used to measure the proliferation and drug sensitivity. Briefly, 10,000 tumor cells were seeded in triplicate in a 96-well plate, and after 2, 24 or 48 hours, the culture medium was replaced with 100 μl of 10% CCK-8 fresh medium. After 2 h of incubation at 37°C, the supernatants were measured spectrophotometrically at 450 nm. In the drug sensitivity assay, 5-fluorouracil (5-FU), doxorubicin (DOX) and cisplatin (DDP) at the concentration of 2 μg/ml or 4μg/ml was added to the medium, whereas no treatment was used as the control.

### Matrigel invasion assay

A cell invasion assay was performed using a 24-well Transwell chamber with a pore size of 8 μm (CoStar, Cambridge, MA, USA). The inserts were coated with 100 μl of matrigel (BD Bioscience, San Jose, CA, USA) diluted 1:2 with serum-free cold DMEM and incubated for 30 minutes at 37°C for gelling. The total growth area in the transwell is approximately 33 mm. The tumor cells (1×10^5^ in 100 μl of serum-free medium) were planted in the upper matrigel chamber and incubated for 20 hours at 37°C. The medium supplemented with 10% FBS was added to the lower chamber as the chemoattractant. The membranes were processed according to the manufacturer's instructions for migration assessment. In brief, the un-migrated cells from the upper side of the membrane were washed and removed. The migrated cells in the lower surface of the membrane were fixed with 4% paraformaldehyde, stained with hematoxylin and dried. The average number of pixels that were positive for cells in randomly chosen fields of view was counted to quantify the extent of invasion using the Photoshop software.

### Experimental animal model

To establish a breast cancer model, 1×10^5^ 4T1-NC or 4T1-GZMM cells were subcutaneously inoculated into the right groin of Balb/C mice or NOD/SCID on day 0 (six to eight mice per group). The tumor growth was monitored every 3-4 days by palpation, and the tumor size was measured through two perpendicular tumor diameters, as described previously. On day 28, the mice were sacrificed, the lungs were resected, and the number of nodules was enumerated. Liver, heart, and renal tissues were isolated to evaluate the change in metastases. In the CT26 mouse model, 2×10^5^ (or 1×10^5^) cells were s.c. inoculated into the dorsal flank of Balb/C mice (five mice per group), and the tumor growth was then detected in a similar manner. In the LLC mouse model, 4×10^5^ cells were s.c. inoculated into the dorsal flank of C57 mice.

### Clone forming assay

300 or 500 cells were seeded in 60mm-plates in quadruplicate with complete medium and cultured for one week at 37°C. After cultured, cells were fixed, stained with Giemsa and counted.

### Western blot

Cells or tumor tissues were collected and suspended in RIPA lysis buffer (Biomiga, Inc.) containing a cocktail of proteinase inhibitors (Roche). The protein concentration was quantified using the bicinchoninic acid (BCA) assay kit (Thermo scientific, Inc.) to ensure that equal amounts of protein from different subpopulations were loaded into the gel. The proteins were separated by SDS-PAGE and transferred onto a polyvinylidene difluoride (PVDF) membrane. Primary antibodies against murine GZMM (P-15, Santa Cruz Biotechnology, Inc.); E-cadherin, N-cadherin, vimentin, AKT, p-AKT, ERK1/2, p-ERK1/2, STAT3, p-STAT3^705^, p-STAT3^727^ (Cell Signaling Technology), a-tubulin (ZSGB-BIO), and β-actin (ZSGB-BIO) were used at 1:1000 dilution and incubated with the membranes at 4°C overnight. The reaction was revealed by horseradish peroxidase (HRP)-coupled secondary reagents (Pierce, Rockford, IL, USA), developed by enhanced chemiluminescence (ECL) (Applygen Technologies Inc.) according to the manufacturer's instructions, and subjected to exposure using LAS4010 (General Electric Company).

### Flow cytometry analysis

Anti-GZMM (LifeSpan BioSciences) or isotype control antibody was used to stained tumor cells or PBMC according to the instruction of intracellular staining kit (BD). FITC-conjunced donkey anti-rabbit IgG antibody (Biolegend) was used as the secondary antibody, and cells were analyzed by a Guava easyCyte flow cytometer (Milipore) and Flowjo software.

### Real-time reverse transcription-polymerase chain reaction

The total RNA from the cultured cells was extracted using the TRIzol reagent (Invitrogen, USA) according to the manufacturer's instructions. The cDNA library was then reverse-transcribed using the iSCRIPT cDNA synthesis kit (Bio-Rad Laboratories, USA) according to the manufacturer's instructions. Quantitative RT-PCR (qRT-PCR) was performed using iUNIVERSAL SYBR Green I Mix kit (Bio-Rad Laboratories, USA) with a CFX96 Real-Time PCR System (Bio-Rad Laboratories, USA) as previously described. The following specific primers were used (Table 1):

**Table 1:** 

murine GZMM (158 bp)	forward	5′-GTCCTTGTGCATCGGAAGTG-3′
	reverse	5′-TTGTAGCCAGGGTGTTTAATGG-3′
murine β-actin (154 bp)	forward	5′-GGCTGTATTCCCCTCCATCG-3′
	reverse	5′-CCAGTTGGTAACAATGCCATGT-3′
murine GZMB (142 bp)	forward	5′-CCACTCTCGACCCTACATGG-3
	reverse	5′-GGCCCCCAAAGTGACATTTATT-3′
murine perforin (272 bp)	forward	5′-CTGGCTCCCACTCCAAGGTA-3′
	reverse	5′-GGCTGTAAGHACCGAGATGC-3′
human GZMM (196 bp)	forward	5′-ACACCCGCATGTGTAACAACA-3′
	reverse	5′-GGAGGCTTGAAGATGTCAGTG-3′
human β-actin (218 bp)	forward	5′-AAGAGAGGCATCCTCACCCT-3′
	reverse	5′-TACATGGCTGGGGTGTTGAA-3′
human perforin (233 bp)	forward	5′-GGCTGGACGTGACTCCTAAG-3′
	reverse	5′-CTGGGTGGAGGCGTTGAAG-3′
human GZMB (118 bp)	forward	5′-GGTGCGGTGGCTTCCTGAT-3′
	reverse	5′-TGCTGGGTCGGCTCCTGTTC-3′

### Statistical analysis

The statistical significance of the difference between the two groups was determined by Student's *t*-test. The Kaplan-Meier survival plot was assessed for significance using the log-rank test (SPSS 12.0). A value of p < 0.05 was considered significant.

## SUPPLEMENTARY MATERIAL FIGURES AND TABLES






