# Supplementary material for: Granzyme M expressed by tumor cells promotes chemoresistance and EMT in vitro and metastasis in vivo associated with STAT3 activation
Source: Oncotarget. 2015 Feb 28;6(8):5818–31. doi: 10.18632/oncotarget.3461 (PMC4467404; doi:10.18632/oncotarget.3461)
Supplement: Supplementary file 1 [file oncotarget-06-5818-s001.pdf]

Granzyme M expressed by tumor cells promotes chemoresistance and EMT *in vitro* and metastasis *in vivo* associated with STAT3 activation

Supplementary Material

Pathway	Count	p-Value	q-Value	Gene
**Cytokine-cytokine receptor interaction**	18	2.07709E-19	4.00879E-17	Kitl; Ccl25; Cxcl1; Ccr1 ;Cxcl5 ;Ccl2 ;Lif; Ccl7; Tnfrsf10b; Il1a; Egfr; Inhbb; Csf2; Cxcl16; Il11; Il6; Fas; Eda2r;
**Jak-STAT signaling pathway**	11	2.95923E-12	1.42783E-10	Stam2; Stat1; Lif; Socs4; Il13ra2; Pim1; Csf2; Il11; Il6; Pik3r5; Pik3r3
**VEGF signaling pathway**	5	3.77328E-06	3.73458E-05	Ptgs2; Hspb1; Mapkapk3;Pik3r5;Pik3r3
**Cell adhesion molecules (CAMs)**	4	0.001571295	0.006034996	Selp; Itgav; Cd80; Cldn9;
**Regulation of actin cytoskeleton**	7	5.00242E-06	4.5732E-05	Itgav; Msn; Enah; Egfr; Bdkrb1; Pik3r5; Pik3r3
**Drug metabolism - other enzymes**	4	2.55785E-05	0.000189871	Tk1;Cyp3a13;2210023G05Rik;Ces5

Figure S1. Several important pathways that are critical for cancer survival, chemoresistance, angiogenesis and inflammation.

Supplementary Table S2. Information of patients with colon cancer.

Variable	No. of patients (%) (n = 90)
Age(years) (mean±SD)	63.5±12.5
Gender	
Male	57 (63.3)
Female	33 (36.7)
Clinical stage	
Ⅰ	17 (18.9)
Ⅱ	23 (25.6)
Ⅲ	35 (38.9)
Ⅳ	15 (16.7)
Histologic type	
Well to moderately differentiated	67 (74.4)
Poorly differentiated to undifferentiated	23 (25.6)
Distant Metastasis	15 (16.7)
Lymphatic Metastasis	46 (51.1)

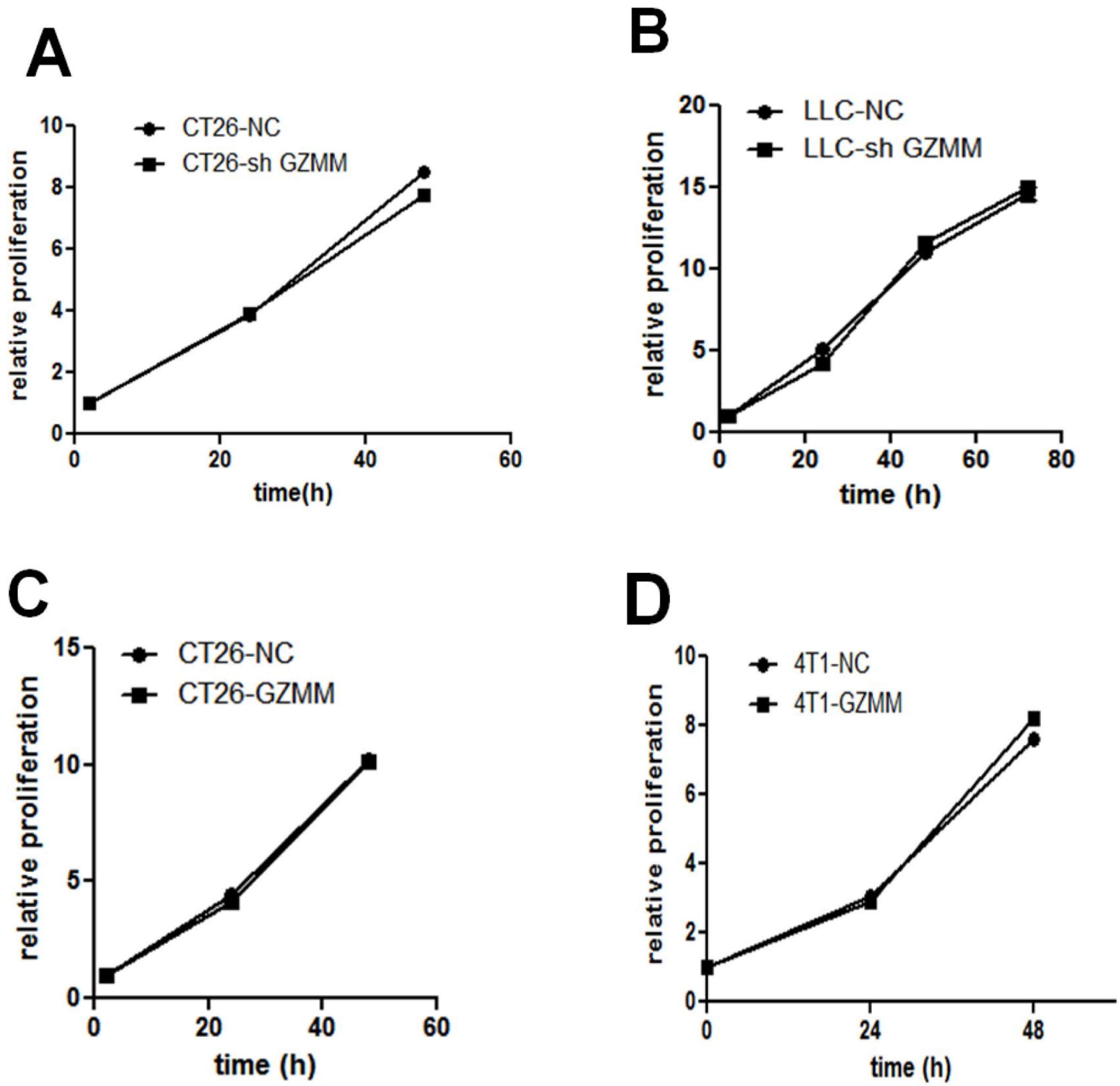

Figure S3. GZMM had almost no significant influence on the proliferation of 4T1, LLC and CT26 cells.
